# The Role of Quantitative and Semi-quantitative [^18^F]FDG-PET/CT Indices for Evaluating Disease Activity and Management of Patients With Dermatomyositis and Polymyositis

**DOI:** 10.3389/fmed.2022.883727

**Published:** 2022-04-15

**Authors:** Halil Yildiz, Philippe D'abadie, Olivier Gheysens

**Affiliations:** ^1^Department of Internal Medicine and Infectious Diseases, Cliniques Universitaires Saint-Luc and Institute of Clinical and Experimental Research (IREC), Université Catholique de Louvain (UCLouvain), Brussels, Belgium; ^2^Department of Nuclear Medicine, Cliniques Universitaires Saint-Luc and Institute of Clinical and Experimental Research (IREC), Université Catholique de Louvain (UCLouvain), Brussels, Belgium

**Keywords:** [^18^F]FDG-PET/CT, dermatomyositis, polymyositis, cancer, interstitial lung disease, standardized uptake value

## Abstract

Idiopathic inflammatory myopathies (IIM) are considered systemic diseases involving different organs and some subtypes are associated with increased cancer risk. In this review, we provide a comprehensive summary of the current use and potential applications of (semi-)quantitative [^18^F]FDG-PET/CT indices in patients with IIM focusing on dermatomyositis and polymyositis. Visual interpretation and (semi-)quantitative [^18^F]FDG-PET indices have a good overall performance to detect muscle activity but objective, robust and standardized interpretation criteria are currently lacking. [^18^F]FDG-PET/CT is a suitable modality to screen for malignancy in patients with myositis and may be a promising tool to detect inflammatory lung activity and to early identify patients with rapidly progressive lung disease. The latter remains to be determined in large, prospective comparative trials.

## Introduction

Idiopathic inflammatory myopathies (IIM), collectively known as myositis, are heterogeneous disorders characterized by muscle inflammation and weakness that can affect children and adults (1). Based on clinical and histopathological features, IIM are classified into four subgroups: dermatomyositis (DM), polymyositis (PM), immune-mediated necrotizing myositis (IMNM) and inclusion body myositis (IBM) (2, 3).

A wide variety of diagnostic or classification criteria for myositis are used since the original Bohan and Peter classification in 1975. This remains the most widely used criteria in IIM to date (4). DM and PM were differentiated based on the presence of the characteristic skin rashes. At the time of the Bohan and Peter classification, a new entity known as inclusion body myositis (IBM) was described showing distinct clinical and laboratory features and was subsequently incorporated in the classification of Dalakas and Hohlfeld (5). Based on the discovery of several myositis-specific antibodies (MSA) in the 1990s and to further increase the specificity of the criteria, Targoff et al. (6) proposed a modified Bohan and Peter criteria by including myositis-specific antibodies (MSA) and MRI findings. In 2002, Sontheimer et al. proposed to expand the spectrum of DM by including (7) hypomyopathic and amyopathic dermatomyositis (ADM) that was incorporated in the 2003 revised Dalakas criteria (5). The criteria of the European Neuromuscular Center (ENMC) published in 2004 required histological confirmation together with clinical and laboratory criteria (8). Later, Troyanov et al. (9) proposed a clinico-serological approach by combining IIM clinical features with auto-antibodies profiles. As outlined above, classification of IIM are generally empirical, lack proper validation and remain a major subject of debate. Therefore, a group of IIM experts was assembled to develop easily applicable classification criteria with limited clinical and laboratory features. The 2017 EULAR/ACR Criteria employs easily and widely available criteria combining clinical, biological and histopathological findings (10). The criteria have been partially validated and seem to perform better than existing criteria but these criteria are intended to classify but not to diagnose the different entities (11).

From a diagnostic perspective, clinical and biological findings (elevation of creatine kinase (CK) levels and presence of autoimmune antibodies) are the cornerstone for diagnosing IIM. Muscle biopsy remains the gold standard to confirm the diagnosis but it is an invasive technique that requires surgical experience (3, 10).

Electromyography and magnetic resonance imaging (MRI) are considered useful to confirm muscle involvement but are not able to differentiate myopathies (3, 10). Magnetic resonance imaging detects inflammatory oedema and is very useful since it may give information on disease activity and help clinicians to select the site of biopsy (12). However, not all proximal muscles are covered by MRI (limited field of view and resolution) and even though whole-body MRI has been validated in pediatric patients (13), the application of whole-body MRI in adult patients is difficult (length of procedure, not widely available, need of an experienced radiologist). Moreover, MRI is not able to make the difference between inflammatory myopathies and other muscle diseases (14) and some patients are not suitable for MRI (e.g., patients with pacemaker or claustrophobia).

[^18^F]FDG-PET/CT is mainly used to exclude malignancy in patients with inflammatory myopathies. Kundrick et al. (15) showed that [^18^F]FDG-PET/CT is a cost effective technique for diagnosing malignancy in the context of DM. However, [^18^F]FDG-PET/CT may also be useful for diagnosing myositis, to evaluate disease extent, to identify the site of biopsy and to exclude the presence of interstitial lung diseases. To date, the role of [^18^F]FDG-PET/CT in patients with inflammatory myositis is not well-defined. The objective of our review is to describe the clinical value of [^18^F]FDG-PET/CT in the diagnosis and management of patients with inflammatory myositis focusing on DM and PM using visual, semi-quantitative and quantitative methods.

## Methodology

A comprehensive literature search through PubMed/MEDLINE databases was carried out until 01 January 2022. The following search algorithm combining several mesh terms was used: [(FDG) OR (fluorodeoxyglucose) OR (PET) OR (positron)] AND [(polymyositis) OR (dermatomyositis) OR (myositis) OR (inflammatory myopath^*^)]. The search was restricted to articles in English language and no other restrictions were applied to the database search.

### Exclusion and Inclusion Criteria

Title and abstract of the retrieved records was independently screened by two reviewers (H.Y. and P.D.A.) based on predefined selection criteria. Inclusion criteria were original research articles on the diagnostic performance of [^18^F]FDG-PET/CT for inflammatory myositis or the use of [^18^F]FDG-PET/CT for assessing malignancy or interstitial lung disease in patients with myositis. Exclusion criteria were review articles, case reports or small case series (<10 patients), comments, editorials or letters to the editor and articles not related to the scope of this review. The content of the selected articles was evaluated before inclusion and those that did not provide sufficient data on the topic were excluded from this review.

### Data Collection

The following data were extracted from the studies and cross-checked by two reviewers (H.Y. and P.D.A.): authors, year of publication, country, study design, number of patients, type of myositis, steroid or other immunosuppressive treatment prior to [^18^F]FDG-PET/CT, reference standard for diagnosis/classification, [^18^F]FDG-PET/CT interpretation criteria, diagnostic performance parameters of [^18^F]FDG-PET/CT, comparison with MRI, electromyography or muscle biopsy if available and other relevant findings.

## Results

### Literature Search, Study and Patient Characteristics

**Figure 1:**
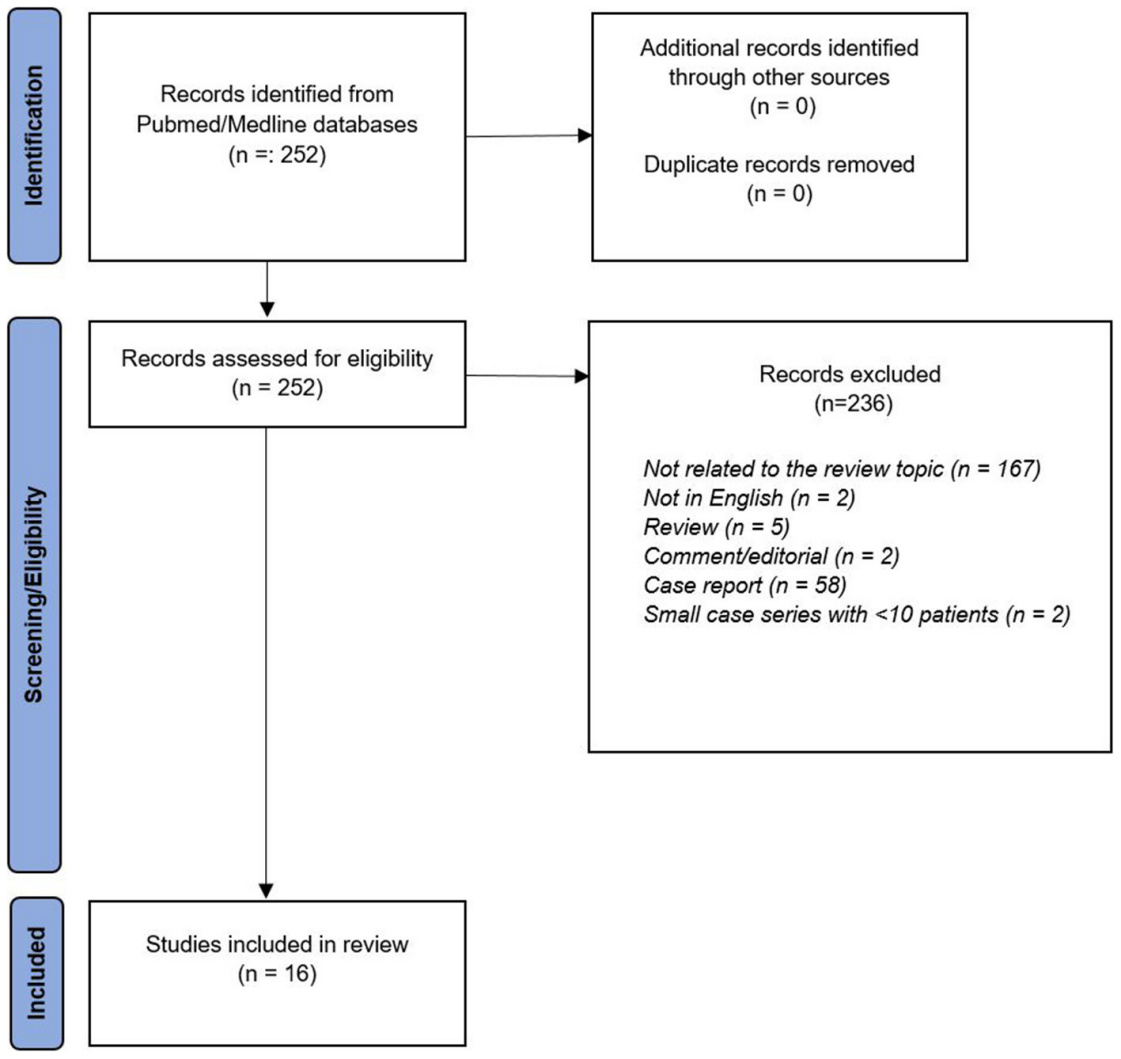
Flow diagram.

The database search identified 252 records found and 236 records were excluded after title/abstract screening and full-text content because those did not provide any relevant information on the topic of this review (Figure 1). Sixteen articles were eligible for a more detailed description in this review (16–31).

A summary of the patient characteristics and main results of the studies are shown in Tables 1–**3**. All studies except two were retrospective in nature (the majority of studies included both DM and PM (13/16) with a DM predominance while 3 studies only included patients with DM. More than half of the studies (9/16) included patients who received corticosteroid treatment before performing [^18^F]FDG-PET/CT. The majority of studies (10/16) included a control group that consisted of cancer patients (pulmonary and melanoma cancer) except in one study in which patients with amyotrophic lateral sclerosis served as control group (20). Different reference standards were used among the studies to classify patients with inflammatory myositis with half of the studies using the Bohan and Peter criteria alone (8/16) or in combination with the Sontheimer criteria (2/16). The other classification criteria used were, respectively, the 2017 ACR EULAR (3/16), ENMC (2/16), and Allenbach (1/16).

**Table 1:** Studies evaluating the performance of [^18^F]FDG-PET/CT in inflammatory myopathies.

**Studies**	**Type of study**	**Patient population and criteria for IM**	**Control patients: indication**	**Myositis on muscle biopsy**	**PET CT interpretation criteria**	**PET-CT diagnostic performance parameters**	**Other findings**	**Incidental findings**
Owada et al. (17)^*^ Country: Japan	Retrospective	13 DM, 11 PM *Bohan Peter criteria*	69: Malignancy	17/17	**Visual analysis**: Positive if FDG accumation was equal or higher than the liver uptake in limbs	Se 33.3%	FDG uptake associated with EMG changes (*p* 0.051) and inflammatory cells into endomysium (p 0.109)	Cancer: 1 ILD: 7/24
Pipitone et al. (18)^*^ Country: Italy	Prospective	10 DM, 2 PM *Bohan Peter criteria*	14: randomly chosen (4: malignancy)	NA	**Semi quantitative:** Average SUVmean Muscle to liver SUVmean ratio	SUV Muscle/liver ratio ≥ 0.45 Se 75%, Sp 100%	No correlation between PET CT uptake and CK level (spearman's *p* > 0.05)	Cancer: 3
Tanaka et al. (19) Country: Japan	Retrospective	15 DM, 5 PM *Bohan Peter criteria*	20: 15 malignancy, 3 benign tumor, 2 inflammatory diseases	19/19	**Semi quantitative analysis**: SUVmean in proximal limb muscles (ROI 20 mm diameter)	SUVmean> 0.83 Se 90%, Sp 100% (AUC 0.95 [95%CI 0.88–1.02])	PET CT performed better than MRI and was correlated with histological findings (*p* 0.009) and correlated with CK level (*P* < 0.05)	ILD: 9/20
Tateyama et al. (20)^*^ Country: Japan	Retrospective	11 DM, 11 PM, 11 OM *Bohan Peter criteria*	22: amyotrophic lateral sclerosis	31/33	**Visual analysis**: positive if FDG uptake greater or equal to mediastinal blood pool **Semi quantitative analysis** (ROI 20 mm diameter): SUVmean, SUVmax, mean SUV mean and mean SUV max of 6 muscle regions	visual analysis: Se 60.6% Patients vs. control mean SUVmean 1.1 (±0.4) vs. 0.7 (± 0.1) mean SUVmax 1.5 (±0.5) vs. 1.0 ±0.1	Comparison of PET CT and MRI findings in 25 patientsMRI positive 20/25; PET positive in the same muscle in only 4/25 No correlation SUVmax and CK level (*p* = 0.08)	Cancer: 3 ILD: 11/33
Li et al. (21)^*^ Country: China	Retrospective	18 DM, 3 PM, 17 ADM *Bohan Peter criteria* *Sontheimer criteria*	22: Malignancy exclusion	NA	**Semi quantitative analysis:** Mean SUV max of limb muscles	Patients vs. control Mean SUVmax 1,6 (±0,9) vs. 0.9 ±0.1	PET was correlated with CK level (*p* = 0.042)	Cancer: 8 ILD: 30/38
Sun et al. (22) Country: China	Retrospective	22 DM/PM *Bohan Peter criteria*	22: patients without myopathy	13/14	**Semi quantitative analysis** SUV max in 7 proximal muscular regions mean SUV max	SUV max ≥ 1.86 Se 95.5%, Sp 95.5%	The average SUVmax in cervical, thoracic, lumbar regions were correlated with CK levels (*p* < 0.05)	Cancer: 1 ILD: 16/22
Matuszak et al. (23)^*^ Country: France	Retrospective	11 DM, 1 PM, 5 OM, 5 NAM *2017 ACR EULAR criteria*	20: malignancy exclusion	NA	**Semi quantitative analysis** Mean SUV max in 16 proximal muscles Ratio mean SUV max/liver SUV mean	Ratio mean SUVmax/liver SUVmean ≥ 0.66 Se 92.3%, Sp 88.9%, 95% CI 74.9–99.1, accuracy 97%	Muscle SUVmax threshold 0.66 allow make difference between active and control patients Se 100%, Sp 92.3%. mSUV max was correlated with CK levels *p* < 0.0001	Cancer: 13
Martis et al. (25)^*^ Country: France	Retrospective	20 DM, 4 ADM *ENMC criteria*	24: melanoma	22/24	**Semi quantitative analysis**: Ratio SUV max proximal muscles/ SUVmax muscle lumbar region	Ratio SUVmax proximal muscles/ SUVmax muscle lumbar region≥1.73 Se 50%, Sp 83.3%	No correlation with CK level	NA
Motegi et al. (26) Country: Japan	Retrospective	22 DM *Bohan Peter criteria* *Sontheimer criteria*	No control	NA	**Visual analysis**: hypermetabolic uptake in muscles	NA	Correlated with CK levels (*p* < 0.05) Myositis diagnosed by MRI (16/22, 73%)	Cancer: 1 ILD: 21/22
Arai-Okuda et al. (28) Country: Japan	Retrospective	18 DM, 10 PM *Bohan Peter criteria*	28: 26 malignancy, 2 inflammatory diseases	NA	**Visual analysis:** positive if FDG uptake greater or equal to mediastinal blood pool (the number of positive regions were calculated in 18 regions–Total score) **Semi quantitative analysis**: Mean SUV mean and mean SUV max in 18 muscle regions	Se 82.1%, Sp 92.9%, AUC 90% (95%CI 0.81–0.99) Total score cut-off: 2 (Se 82.1%, Sp 96.4%, AUC 0.90 [95% CI 0.81–0.99]) Cut-off mean SUVmean: 0.93 Se 78.6%, Sp 89.3%, AUC 82.3% 95% CI 0.70–0.94 Cut-off mean SUVmax: 1.12 Se 85.7%, Sp 96.4%, AUC 88.9% (95% CI 0.79–0.99)	Total score, mean SUV and SUVmax were correlated with CK level (*p* < 0.05)	NA

*IM, inflammatory myopathies; DM, dermatomyositis; PM, polymyositis; OM, overlap myositis; ADM, amyopathic dermatomyositis; NAM, Necrotizing autoimmune myositis; IBM, inclusion body myositis; ILD, interstitial lung diseases; RP-ILD, rapidly progressive interstitial lung diseases; HRCT, high-resolution computed tomography; CK, creatine kinase; vFDG, visually identified FDG uptake; SUV, standardized uptake values; SUV prox/mlt, SUV in proximal muscles/musculus longissimus thoracis; MDA5, melanoma differentiation-associated gene 5*;

* *corticosteroid given prior to PET/CT; ENMC, European Neuromuscular Center; ROI, region of interest*.

### Diagnostic Performance of [^18^F]FDG-PET/CT in Inflammatory Myositis

**Figure 2:**
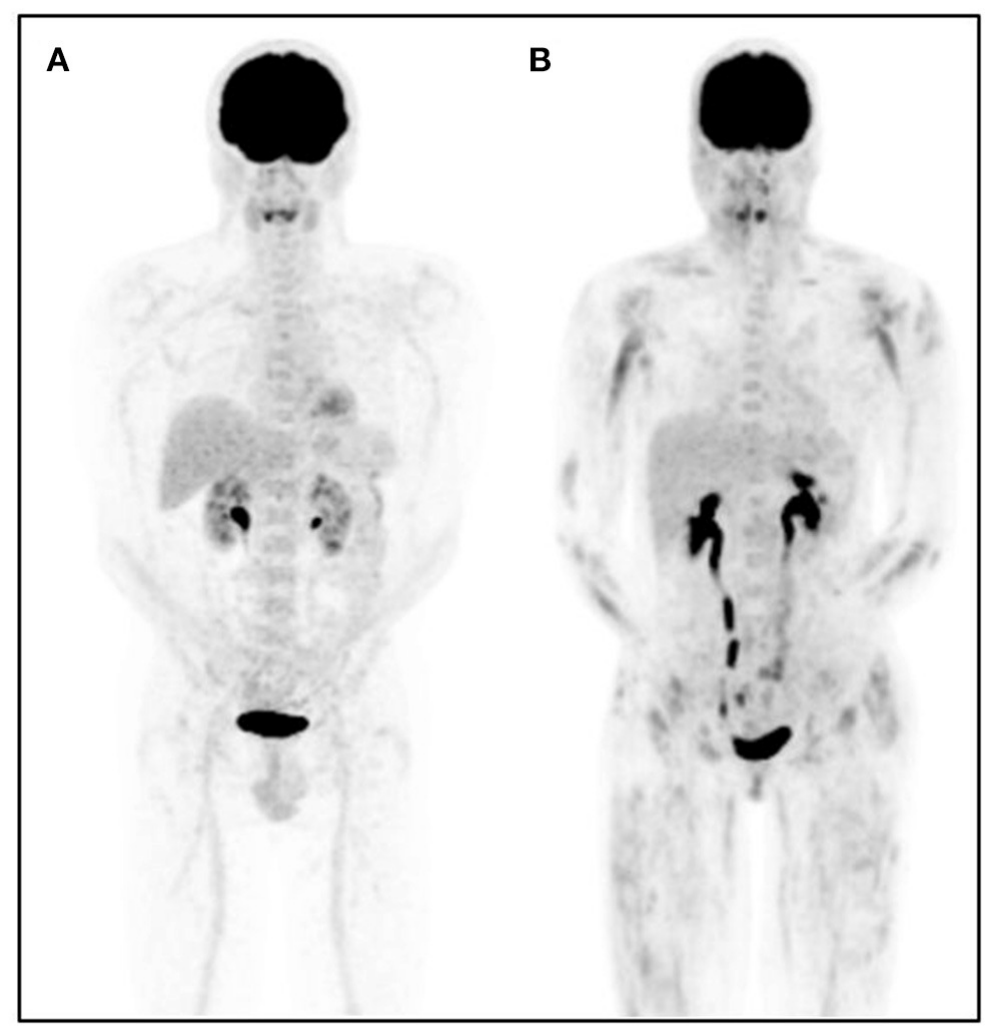
Representative [^18^F]FDG-PET images in a control patient and a patient with dermatomyositis. [^18^F]FDG-PET maximum intensity projection image of a subject with physiologic [^18^F]FDG biodistribution **(A)** and a representative patient with dermatomyositis **(B)**. Proximal and symmetrical heterogeneous [^18^F]FDG muscle uptake is observed in patient **(B)**.

The main results of the 10 studies evaluating the role of [^18^F]FDG-PET/CT to detect disease activity in inflammatory myositis are summarized in Table 1 and a representative image is shown in Figure 2. Different [^18^F]FDG-PET/CT interpretation criteria were used across the studies including a (semi-)quantitative approach in 6 studies, a pure visual/qualitative assessment in 2 studies, and both a visual and semi-quantitative evaluation in 2 studies.

#### Visual Assessment

Two studies compared [^18^F]FDG muscle uptake to mediastinal bloodpool activity with an uptake equal or higher than mediastinal blood pool being considered positive (20, 28). Tateyama et al. (20) used a 2-point grading scale (0 = uptake < mediastinal blood pool and 1 = ≥ mediastinal blood pool) which was evaluated in 16 regions resulting in a composite score ranging from 0 to 16. Muscular [^18^F]FDG uptake (at least 1 point) was observed in 60% of patients while 42% of patients showed multiple affected regions with various patterns, but in a symmetrical distribution. Twenty-five patients underwent both [^18^F]FDG-PET and MRI. In this subgroup, 20 patients were judged MRI positive and concordant PET -MRI findings were observed in only 4/25 patients while none of the MRI negative patients showed pathological muscle uptake on [^18^F]FDG-PET.

The study by Arai-Okuda et al. (28) assessed 18 muscle regions using a 3-point visual scoring system (0 = uptake < mediastinal blood pool, 1 = ≥ mediastinal blood pool and 2 = ≥ liver uptake) resulting in a total score ranging from 0 to 36. Increased [^18^F]FDG uptake in at least one region was observed in 23/28 (82%) patients with IM compared to 2/28 (7%) in the control group. ROC analysis identified an optimal cut-off score of 2 to accurately discriminate IIM patients from control patients with a sensitivity of 82% and specificity of 96%.

Another study applied a 3-point visual grading system using physiological liver uptake as reference (grade 1 = uptake < liver, grade 2 = uptake equal to liver, grade 3 = uptake > liver uptake) with a visual grade 2 or 3 being considered positive for myositis (17). The sensitivity of [^18^F]FDG-PET/CT to detect muscle involvement in 24 patients with IIM was 33% which was significantly lower compared to EMG (73%), MRI (57%) and muscle biopsy (100%).

#### (Semi-) Quantitative Parameters

[^18^F]FDG-PET/CT has now become the standard in modern imaging, allowing developing semi-quantitative and quantitative approaches such as SUVmax, mean SUVmax or SUVratios.

Most studies reported the muscle SUVmean or SUVmax by itself, i.e., the absence of a reference organ. Two studies used the SUVmean of muscle uptake with a cut-off value of 0.83 and 0.93 with sensitivity and specificity of 90, 79, 100, and 89%, respectively (19, 28). The study by Sun et al. (22) reported a cut-off value for SUVmax of 1.86 resulting in a sensitivity and specificity of 96%. A similar specificity but somewhat lower sensitivity (86%) was obtained in the study by Arai-Okuda et al. (28) using a cut-off value of 1.12. A direct comparison between SUVmax and SUVmean values demonstrated a slightly better diagnostic accuracy using a SUVmax cut-off value. Two studies used a ratio of SUV's with liver uptake as reference with optimal cut-off values of 0.45 and 0.66 yielding a sensitivity of 75% and 92% and a specificity of 100 and 89%, respectively (18, 23). The study by Martis et al. (25), evaluating the ratio of muscle uptake in limbs compared to the muscle uptake in the lumbar region, reported a moderate diagnostic accuracy with sensitivity of 50% and specificity of 83% using a cut-off value of 1.73. Finally, a head-to-head comparison between visual interpretation and semi-quantitative (SUVmax) indices in one study demonstrated a similar performance with AUC values of 0.90 and 0.89, respectively.

Additionally, 9 studies investigating the correlation between [^18^F]FDG muscle uptake and serum creatine kinase (CK) levels showed discrepant results with 6 studies (19, 21–23, 26, 28) demonstrating a statistically significant correlation while three other studies (18, 20, 25) did not show any correlation at all.

### [^18^F]FDG-PET/CT and Cancer Diagnosis

Four studies (16, 24, 27, 31) specifically investigated the value of [^18^F]FDG-PET/CT to identify occult malignancy in patients with inflammatory myopathies (IM). The main results are summarized in Table 2. Overall, all studies reported a similarly high negative predictive value ranging between 94 and 98% while specificities ranged between 79 and 98%. In contrast, large ranges in sensitivity (67–94%) and positive predictive value (42–86%) were shown across studies. The study by Maliha et al. (24) showed that [^18^F]FDG-PET/CT failed to detect malignancy in comparison to conventional screening in all patients (*n* = 3). However, it is noteworthy to mention that the cancers were multiple myeloma, squamous cell carcinoma of the skin and small breast cancer, lesions that are either poorly FDG-avid or have a size below the spatial resolution of the camera. In addition, occult malignancies have also been diagnosed using [^18^F]FDG-PET/CT in the majority of other studies performed in IIM patients (Tables 1, 3).

**Table 2:** Studies evaluating the performance of [^18^F]FDG-PET/CT for cancer diagnosis in inflammatory myopathies.

**Studies**	**Type of study**	**Type of IM and criteria for IM diagnosis**	**Gold standard for cancer screening and incidence**	**Performances of PET/CT for cancer screening**
Selva-O' Callaghan et al. (16)^*^ Country: Spain	Prospective	49 DM, 6 PM *Bohan Peter criteria*	Physical examination, laboratory tests, thoracoabdominal CT scan, gynecologycal exams and mammography Incidence: 9 patients (16%)	Se 67%, Sp 98% PPV: 86%, NPV: 94%
Maliha et al. (24) Country: Canada	Retrospective	31 DM, 1 PM, 25 OM, 1 IBM, 5 unspecified *Allenbach criteria*	Physical examination, laboratory tests, thoraco-abdominal CT scan, endoscopies, gynecologycal exams and mammography Incidence: 3 patients (5%)	Se: 0%, Sp: 85% PPV: 0%, NPV: 94%
Li and Tan (27) Country: China	Retrospective	75 DM *Bohan Peter criteria*	No gold standard One false negative result at follow-up (breast cancer) Incidence: 17 patients (23%)	Se 94%, Sp 95% PPV 84%, NPV 98%
Trallero-Araguas et al. (31) Country: Spain	Retrospective	*44 DM, 14 NAM, 4 PM, 16 OM* *ENMC criteria*	Conventional screening including physical examination, thoraco ab gynecologycal exams and mammography dominal CT scan, MRI, endoscopies… Incidence: 11 patients (14%)	Se 91%, Sp 79%, PPV 42%, NPV 98%

*IM, inflammatory myopathies; DM, dermatomyositis; PM, polymyositis; OM, overlap myositis; ADM, amyopathic dermatomyositis; NAM, Necrotizing autoimmune myositis*.

* *Corticosteroid given prior to PET/CT*.

**Table 3:** Studies evaluating the performance of [^18^F]FDG-PET/CT for diagnosis of interstitial lung disease (ILD) associated to inflammatory myopathies.

	**Type of study**	**Population and Criteria for IM diagnosis**	**PET Criteria for ILD**	**Gold standard for ILD diagnosis and incidence**	**ILD diagnosis using PET/CT**	**Semi quantitative analysis**	**Incidental findings**
Owada et al. (17)^*^ Country: Japan	Retrospective	13 DM, 11 PM Bohan Peter Criteria	Visual analysis (increased FDG uptake in the lungs)	HRTC 18 patients (75%)	7 patients (Se: 39%)	No	1 patient with cancer
Tanaka et al. (19) Country: Japan	Retrospective	15 DM, 5 PM Bohan Peter Criteria	Visual analysis (increased FDG uptake in the lungs)	HRCT 9 patients (45%)	5 patients (Se: 56%)	no	1 patient with cancer
Tateyama et al. (20)^*^	Retrospective	11 DM, 11 PM, 11 OM Bohan Peter Criteria	Visual analysis (increased FDG uptake in the lungs, superior to the mediastinum blood vessels)	NA	11 patients (Se: NA)	no	3 patients with cancer
Li et al. (21)^*^ Country: China	Retrospective	18 DM, 3 PM, 17 ADM Bohan Peter Criteria Sontheimer criteria	Visual analysis (increased FDG uptake in the lungs)	HRCT 30 patients (79%) Clinical outcome defined RP-ILD in 7 patients (18%)	28 patients with ILD (Se: 93%) 7 patients with RP- ILD (Se: 100%)	ILD lesions with a mean SUVmax: 2.1 (range 0.9–4.7) RP-ILD: SUVmax ≥ 2.4 (Se: 100%, Sp: 87%)	8 patients with cancer
Motegi et al. (26) Country: Japan	Retrospective	22 DM Bohan Peter Criteria Sontheimer criteria	SUV max in lung lesions	HRCT 21 patients (95%)	21 patients with ILD (Se: 100%)	Significant positive correlation between the lung severity score (HRCT) and SUVmax	1 patient with cancer
Liang et al. (29)^*^ Country: China	Retrospective	40 DM, 9 PM, 12 ADM 2017 ACR EULAR criteria	SUV mean in lung lesions (R0I 20 mm diameter)	HRCT 61 patients (100%) Clinical outcome defined RP-ILD in 21 patients (34%)	61 patients with ILD (Se: 100%) 21 patients with RP-ILD (Se: 100%)	SUVmean >0.45 for RP- ILD (Se 95%, Sp 63%)	7 patients with cancer
Cao et al. (30)^*^ Country: China	Retrospective	26 DM (anti MDA 5 +) 43 DM (anti MDA 5–) *2017 ACR EULAR criteria*	SUV max in lung lesions (ROI 20 mm diameter)	HRCT 25 anti MDA 5+ (96%) 36 anti MDA 5– (84%) Clinical outcome defined RP-ILD in 11 patients (42%) anti MDA 5+ and in 10 patients (23%) anti MDA 5–	NA	Patients anti MDA + had a significant higher SUVmax in lung lesions, in spleen and bone marrow compared to patients anti MDA–	11 patients with cancer Spleen SUV max correlated with RP- ILD and short-term outcome

*IM, inflammatory myopathies; DM, dermatomyositis; PM, polymyositis; OM, overlap myositis; ADM, amyopathic dermatomyositis; MDA5, melanoma differentiation-associated gene 5; ILD, interstitial lung diseases; RP-ILD, rapidly progressive interstitial lung diseases; HRCT, high-resolution computed tomography; SUV, standardized uptake value; ROI, region of interest; NA, no*.

* *Corticosteroid given prior to PET/CT*.

### [^18^F]FDG-PET/CT and Interstitial Lung Diseases (ILD)

Among 16 included studies on inflammatory myopathies, seven studies (17, 19–21, 26, 29, 30) provided information on the value of [^18^F]FDG PET/CT to detect ILD while 3 studies evaluated its role in evaluating disease activity and predictive value. Two studies (26, 29) demonstrated a good concordance between [^18^F]FDG-PET/CT and HRCT to detect ILD while 1 study (17) showed that HRCT better identified ILD patients (18/24 vs. 7/24). Three studies (21, 29, 30) evaluated the performance of semi-quantitative [^18^F]FDG-PET indices to predict rapidly progressive lung diseases (RP-ILD). Li et al. (21) showed that a lung mean SUVmax cut-off ≥2.4 predicted RP-ILD with sensitivity 100% and specificity 87% (accuracy; 90%). Similarly, the study by Liang et al. (29) reported a similarly high sensitivity (95%) with lower specificity (63%) to predict RP-ILD using a lung SUVmean cut-off > 0.454 (AUC 81%). The study by Cao et al. (30) compared semi-quantitative [^18^F]FDG-PET parameters between MDA5+ DM and MDA5- DM patients. Patients with MDA5+ DM had statistically significant higher SUVmax values in lungs, spleen and bone marrow (*p* < 0.05) and spleen SUVmax correlated with RP-ILD and mortality.

## Discussion

This review provides a comprehensive summary on the value of [^18^F]FDG-PET/CT for assessing disease activity and management of patients with dermatomyositis and polymyositis.

Overall, the majority of studies showed that [^18^F]FDG-PET/CT has a good performance for detecting disease activity. Moreover, [^18^F]FDG-PET/CT has proven its value to screen for occult malignancy and may be an added value to assess ILD activity in patients with IIM.

Current data indicate that [^18^F]FDG-PET/CT has an overall good performance to detect muscle activity in patients with IIM but due to differences in methodological aspects and interpretation criteria of [^18^F]FDG-PET/CT, objective interpretation criteria remain to be elucidated. A recent meta-analysis (32) including 4 studies reported a pooled sensitivity of 94% and a pooled specificity of 90% to detect active disease, but considerable heterogeneity in specificity was observed mainly due to differences in methodological aspects and patient characteristics (e.g., treatment with corticosteroids). Even though interpretation criteria were not a source of heterogeneity in this meta-analysis, various interpretation criteria have been proposed and used ranging from a pure visual analysis over semi-quantitative ratios and SUVs. To date, there is no evidence to prefer one method over the other and the only study performing a direct head-to-head comparison between visual analysis and mean SUVmax revealed a similar diagnostic accuracy (28). From a clinical point of view, a visual analysis might be preferred since quantitative strategies usually require a rigorous application of the methodology (e.g., draxing regions of interest) which is time consuming and operator dependent. Moreover, another reason to refrain from SUV-based methods in clinical practice is linked to the inappropriate use of literature reported cut-off values with their respective diagnostic accuracy for detecting muscle activity. Efforts should be made to standardize [^18^F]FDG-PET/CT patient preparation, acquisition protocols and interpretation criteria in patients with IIM similar to other pathologies (33, 34).

Several studies have correlated [^18^F]FDG uptake with serum CK levels as an indicator of disease activity in IIM (18–23, 25, 26, 28). Discrepant results have been observed, but a significant correlation was observed especially when proximal muscle areas were analyzed. Another important message emerged from the study by Tanaka et al. (19) who demonstrated a significant correlation between [^18^F]FDG uptake and histological findings of muscle biopsy suggesting that [^18^F]FDG is a suitable technique to guide for a representative biopsy site. Although there is evidence that [^18^F]FDG is an overall suitable modality to assess disease activity in IIM, few data exists on its usefulness to monitor disease activity over time. The largest evidence could be derived from the study by Matuszak et al. (23) who showed changes in [^18^F]FDG uptake at consecutive time points in 10 patients using the muscle SUVmax/liver SUVmean ratio which correlated well with the clinical muscle disease activity. Finally, though magnetic resonance imaging remains the most widely used technique to evaluate muscle activity in daily practice, the major question remains if [^18^F]FDG-PET/CT (given its current lack of standardized interpretation criteria) has any possible advantage over MRI in assessing disease activity (35).

It is well-known that the risk of cancer is increased in patients with DM and PM and the presence of an occult malignancy is always a concern for the treating physician (36). In addition to clinical assessment and auto-antibody screening, [^18^F]FDG-PET/CT seems a valuable tool for screening malignancy in this population as illustrated in this review. All studies revealed a high NPV and the study by Selva-O'Callaghan et al. (16) reported a similar performance to detect malignancy between [^18^F]FDG-PET/CT and a conventional work-up, with the inherent advantage of the former technique being a one-stop shop modality. However, the use of [^18^F]FDG-PET/CT as screening exam has been matter of debate in terms of cost and radiation exposure, but this has been countered by the study of Kundrick et al. (15) who demonstrated that the cost of [^18^F]FDG-PET/CT was higher for the insurance companies but lower for the patients. Nevertheless, a recent retrospective study by Mihali et al. demonstrated that [^18^F]FDG-PET/CT did not reveal any of the 3 occult cancers that were diagnosed by conventional work-up. As mentioned, the cancers not detected on [^18^F]FDG were multiple myeloma, squamous cell carcinome of the skin and small breast cancer, lesions that are either poorly FDG-avid or had a size below the spatial resolution of the camera. Therefore, more prospective and comparative data are needed on the efficacy of [^18^F]FDG-PET/CT in detecting occult malignancy in patients with IIM.

Since IIM are systemic diseases, other organs can be affected and ILD remains an important cause of mortality in patients with IIM (37, 38). Several groups have demonstrated the possible role of [^18^F]FDG-PET/CT to detect and assess activity of ILD (19–21, 26, 29, 30). The majority of the studies showed a good performance to detect inflammatory lung lesions with a correlation between [^18^F]FDG-PET uptake and lesions on CT. Interestingly, two studies demonstrated that quantitative [^18^F]FDG-PET indices in the lung were predictive of rapidly-progressive ILD (21, 29). These findings suggest that [^18^F]FDG-PET/CT may be useful to early detect inflammatory lesions and to identify those patients at risk for RP-ILD, but large prospective and well-designed trials are needed to elucidate the role of [^18^F]FDG-PET/CT in this setting.

As outlined above, IIM are systemic diseases affecting different organs that are also associated with an increased cancer risk which could benefit from a holistic approach using a whole-body [^18^F]FDG-PET-CT technique enabling to evaluate both inflammatory disease activity and occult cancer. The latter has been exemplified by several studies described in this review. However, objective and robust interpretation criteria have yet to be determined and well-designed prospective trials may provide an answer to the value of [^18^F]FDG-PET/CT in the work-up of patients with ILD.

Our study has several limitations. We performed a critical review of the existing literature without performing a systematic review or meta-analysis. It is well known that muscular [^18^F]FDG uptake can be seen in a variety of conditions such as functional muscle activation due to strenuous exercise, imperfect fasting or insulin resistance. As such, both visual and semi-quantitative parameters are highly dependent on optimal and standardized patient preparation protocols, which makes it difficult to compare results across the studies included. Therefore, it is needless to mention that standardized patient preparation and interpretation criteria in well-designed prospective trials are required to elucidate the value of [^18^F]FDG-PET/CT for diagnosing IIM.

## Conclusion

[^18^F]FDG-PET/CT has a good overall performance to detect and evaluate disease activity in patients with IIM, but objective and robust interpretation criteria have yet to be determined. In addition, [^18^F]FDG-PET/CT has proven useful to screen for occult malignancy and may be a promising tool to assess and monitor ILD extent and activity, and to early identify patients at risk for rapidly progressive ILD. As such, [^18^F]FDG-PET/CT is a valuable imaging tool allowing a multi-organ assessment in a one-stop shop approach. Future well-designed prospective trials may provide an answer to further elucidate the usefulness of [^18^F]FDG-PET/CT in the work-up of ILD.

## Author Contributions

HY and PD'a performed the literature screening. HY, PD'a, and OG contributed to the design and the writing of the manuscript. All authors contributed to the article and approved the submitted version.

## Conflict of Interest

The authors declare that the research was conducted in the absence of any commercial or financial relationships that could be construed as a potential conflict of interest.

## Publisher's Note

All claims expressed in this article are solely those of the authors and do not necessarily represent those of their affiliated organizations, or those of the publisher, the editors and the reviewers. Any product that may be evaluated in this article, or claim that may be made by its manufacturer, is not guaranteed or endorsed by the publisher.
